# Tripolin A, a Novel Small-Molecule Inhibitor of Aurora A Kinase, Reveals New Regulation of HURP's Distribution on Microtubules

**DOI:** 10.1371/journal.pone.0058485

**Published:** 2013-03-13

**Authors:** Iliana A. Kesisova, Konstantinos C. Nakos, Avgi Tsolou, Dimitrios Angelis, Joe Lewis, Aikaterini Chatzaki, Bogos Agianian, Athanassios Giannis, Maria D. Koffa

**Affiliations:** 1 Department of Molecular Biology and Genetics, Democritus University of Thrace, Alexandroupolis, Greece; 2 Chemical Biology Core Facility, European Molecular Biology Laboratory, Heidelberg, Germany; 3 Department of Medicine, Democritus University of Thrace, Alexandroupolis, Greece; 4 Institute for Organic Chemistry, University of Leipzig, Leipzig, Germany; University of Saarland Medical School, Germany

## Abstract

Mitotic regulators exhibiting gain of function in tumor cells are considered useful cancer therapeutic targets for the development of small-molecule inhibitors. The human Aurora kinases are a family of such targets. In this study, from a panel of 105 potential small-molecule inhibitors, two compounds Tripolin A and Tripolin B, inhibited Aurora A kinase activity *in vitro*. In human cells however, only Tripolin A acted as an Aurora A inhibitor. We combined *in vitro*, *in vivo* single cell and *in silico* studies to demonstrate the biological action of Tripolin A, a non-ATP competitive inhibitor. Tripolin A reduced the localization of pAurora A on spindle microtubules (MTs), affected centrosome integrity, spindle formation and length, as well as MT dynamics in interphase, consistent with Aurora A inhibition by RNAi or other specific inhibitors, such as MLN8054 or MLN8237. Interestingly, Tripolin A affected the gradient distribution towards the chromosomes, but not the MT binding of HURP (Hepatoma Up-Regulated Protein), a MT-associated protein (MAP) and substrate of the Aurora A kinase. Therefore Tripolin A reveals a new way of regulating mitotic MT stabilizers through Aurora A phosphorylation. Tripolin A is predicted to bind Aurora A similarly but not identical to MLN8054, therefore it could be used to dissect pathways orchestrated by Aurora kinases as well as a scaffold for further inhibitor development.

## Introduction

Temporal and spatial coordination of the process of mitosis and cytokinesis is a prerequisite for accurate and equal segregation of genomic and cytosolic material into two daughter cells. Among the network of regulatory proteins, Aurora kinases are of particular importance. In terms of enzymatic activity, Aurora kinases belong to the Ser/Thr kinase family and they comprise of two domains: a regulatory domain at the NH_2_-terminus and a catalytic domain at the COOH-terminus. Auroras share a great degree of homology in their catalytic domain, whereas differ in their NH_2_-terminal domain. The mammalian orthologs are at least three: Aurora A, Aurora B and Aurora C [1].

By means of phosphorylating different substrates, including TPX2 [2], Ajuba [3], TACC3 [4], [5], Eg5 [6] and HURP [7], [8] among others, Aurora A is implicated in diverse cell cycle events: centrosome maturation and separation, mitotic entry, bipolar spindle assembly, chromosome alignment, spindle checkpoint and cytokinesis. TPX2 is not merely a substrate but also the best-studied activator of Aurora A, required for Aurora A localization to spindles [2]. Moreover, Aurora A regulates the mitotic spindle apparatus *in Xenopus* as part of a multi-protein complex along with the kinesin Eg5 and three MAPs; TPX2, XMAP215 and HURP [9]. HURP is a MT stabilizer with distinct features since it localizes mainly to kinetochore MTs (kt-MTs) of the mitotic spindle [9], [10] and induces a unique MT conformation *in vitro* [11]. Previous studies suggested a regulatory mechanism where phosphorylation of HURP by Aurora A controls its MT binding [8], [12].

Aurora A is frequently amplified and/or over-expressed in diverse tumor types [13], while over-expression of Aurora A is associated with aneuploidy, centrosomal abnormalities [14], [15] and linked to chromosomal instability [16], features that play key roles in tumor progression. Cells that overexpress Aurora A exhibit substantial resistance to Taxol-induced apoptosis, a common MT targeted chemotherapeutic drug [17].

Small-molecule inhibitors of Aurora kinases are expected to prevent the continuous growth of cancer cells and control abnormal mitosis. Consequently, special interest has been arisen in developing Aurora-specific small-molecule inhibitors that block its activity and function in targeted cancer chemotherapeutics [18], [19]. A growing number of Aurora kinase inhibitors have been developed, including VX-680 [20], MLN8054 [21], [22], and MLN8237 [23], TC28 [24], Hesperadin [25], ZM-447439 [26], [27], PHA-680632 [28].

Although all three Aurora kinases share high sequence similarities at the kinase domain some small differences do exist that can be exploited for the development of such specific inhibitors. Here we describe the development of a novel potent Aurora A inhibitor, named Tripolin A, and report its effect on cultured human cells. Our results indicate that Tripolin A inhibits Aurora A kinase but not Aurora B, in mammalian cells, while it is used to reveal a new way of regulating the function of its substrates, i.e. by altering the distribution of HURP on spindle MTs. Considering the plethora of pathways and the diversity of protein complexes that Auroras participate, Tripolin A could be used to dissect their role in interphase and mitosis.

## Results

### Tripolins inhibit Aurora kinase activity *in vitro*

**Figure 1:**
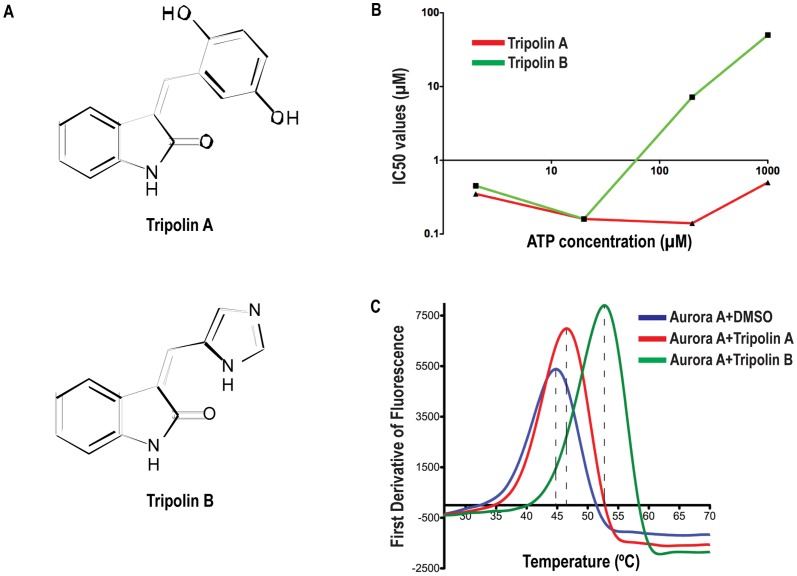
Tripolins inhibit Aurora kinase activity *in vitro*. *(*A) Chemical structure of Tripolin A and Tripolin B. (B) Graph showing IC_50_ values (in µM) of Tripolin A (red) and Tripolin B (green) in the presence of different ATP concentrations, using an *in vitro* kinase assay. (C) Differential Scanning Fluorimetry results for Aurora A in the presence and absence of the inhibitors. Blue curve determines the melting temperature of Aurora A alone (45°C), red in the presence of Tripolin A (47°C) and green in the presence of Tripolin B (53°C).

A library of 105 ATP-analogues was synthesized and their activity against Aurora A using two *in vitro* kinase assays was determined. Two compounds (OXVW5 and OXVW25) showing an inhibition greater than 70%, at a concentration of 10 µM were further investigated and hereafter referred to as Tripolin A and Tripolin B, respectively (Figure 1A).

The effects of increasing concentrations of ATP on the inhibitory activity of the two compounds were examined using *in vitro* kinase assays. The IC_50_ value of Aurora A inhibition by Tripolin B was found to increase with increasing concentrations of ATP present in the reaction (Figure 1B), consistent with an ATP-competitive mode of inhibition, although the competition was apparent only in higher concentrations of ATP (more than 200 µM). Tripolin's A inhibition on Aurora A kinase activity however, remained unchanged in the presence of increasing ATP concentrations (Figure 1B), suggesting that Tripolin A acts as a non ATP-competitive inhibitor.

Selective inhibition of Tripolins against Aurora A was investigated using Aurora B and a panel of receptor tyrosine kinases (Table 1). Despite the relatively limited specificity of Tripolins for Aurora A *in vitro*, the fact that two similar small-molecule compounds showed ATP competitive and non-competitive mode of action prompted us to investigate them further.

**Table 1:** Selectivity of Tripolins against a panel of kinases.

	IC_50_ values (µM)	
Kinase analyzed	Tripolin A	Tripolin B
Aurora A	1.5	2.5
Aurora B	7.0	6.0
EGFR	11.0	71.7
FGFR	33.4	38.0
KDR	17.9	6.5
IGF1R	14.9	13.2

IC_50_ values of Tripolin A and Tripolin B against Aurora A, Aurora B and a panel of other selected kinases.

We examined the relative binding strength of Tripolins to Aurora A by performing differential scanning fluorimetry (DSF) [29], where binding affinities are measured indirectly as a function of the protein's melting temperature (T_m_) increment. Although both Tripolins bound Aurora A, they exhibited differential affinity (Figure 1C). In the absence of the small-molecules the T_m_ of Aurora A, determined from the protein-unfolding midpoint, was found to be 45°C. The presence of Tripolin A induced a change of the unfolding transition temperature (ΔT_m_) of 2°C, while the presence of Tripolin B resulted into a much higher ΔT_m_ (8°C), apparently stabilizing better the Aurora A kinase. Since the difference between the ΔT_m_ values is related to the binding affinity of the small-molecules, these data indicate that Tripolins recognize different binding sites on Aurora A.

### Tripolin A reduces active Aurora A kinase *in vivo*

Phosphorylation at Thr-288 within the activation loop (A-loop) is necessary for Aurora A kinase activity [30]. Hence, the effect of Tripolins on Aurora A in mammalian cells was evaluated by immunofluorescent detection of Aurora A auto-phosphorylation on T288.

**Figure 2:**
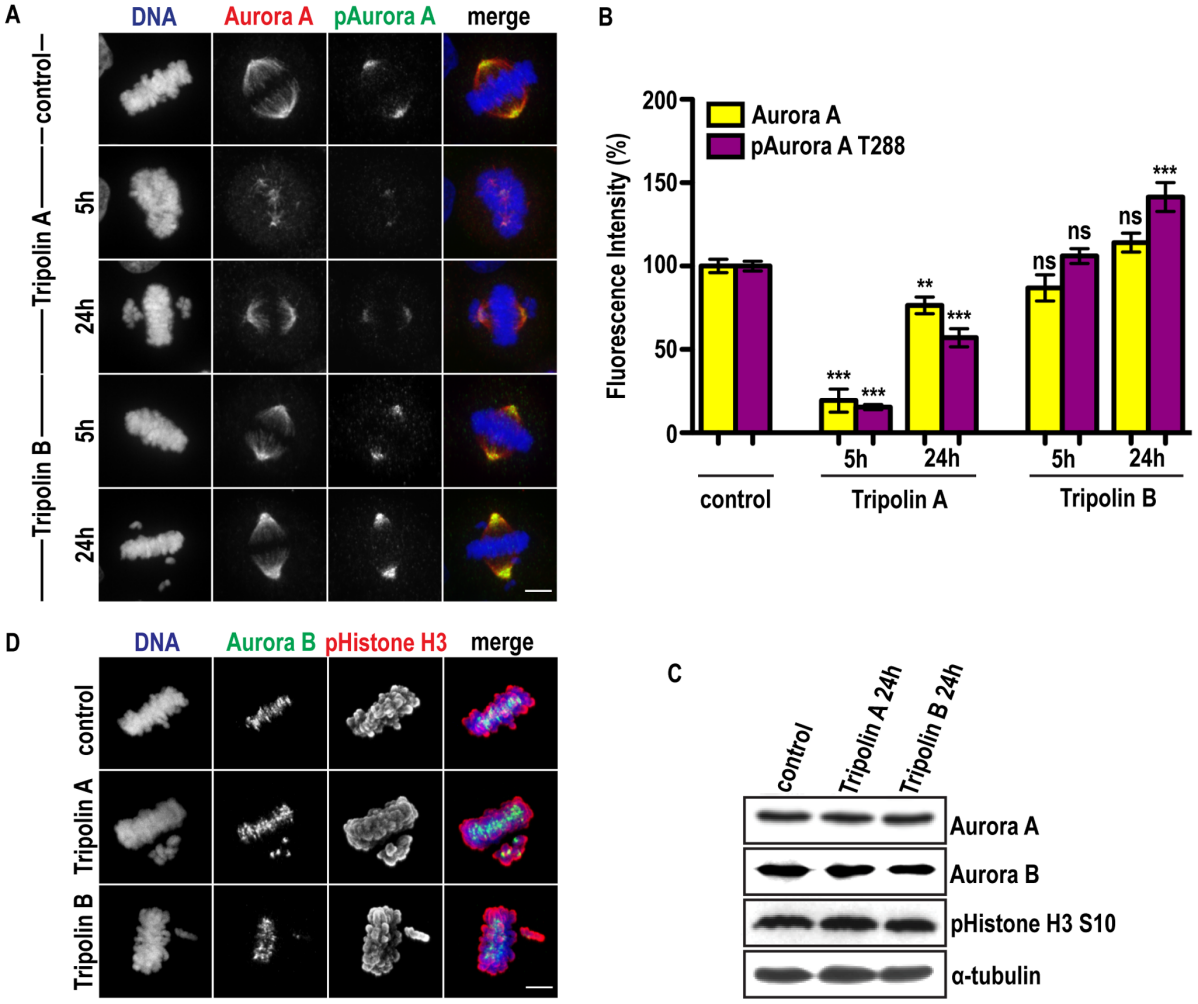
Tripolin A selectively inhibits Aurora A over Aurora B in cultured tumor cells. (A) Representative immunofluorescence images of HeLa cells in metaphase treated with solvent control (DMSO), 20 µM Tripolin A or Tripolin B for 5 h and 24 h. In the merged images Aurora A is pseudocolored red, pAurora T288 green, DNA blue. (Scale bars, 5 µm). (B) Fluorescence intensity (% percentage) of pAurora A T288 on centrosomes and total Aurora A on spindles were quantified in control metaphase cells or cells treated with Tripolin A or Tripolin B (n≥20 cells for each group, from at least two independent experiments). **: 0.001<p<0.01; ***: p<0.001; ns: p>0.05; (Mann-Whitney test, two-tailed). Error bars represent SEM. (C) Western Blot analysis for Aurora A, Aurora B and pHistone H3 Ser10 in Tripolin A and Tripolin B-treated mitotic cells. α-tubulin was used as a loading control. (D) Representative immunofluorescence images of bipolar metaphase HeLa cells treated with solvent control (DMSO), 20 µM Tripolin A or Tripolin B for 24 h. In the merged images pHistone H3 Ser10 is pseudocolored red, Aurora B green, DNA blue. (Scale bars, 5 µm).

In control (DMSO-treated) cells, pT288 was detected only in mitotic cells and its localization was restricted on centrosomes. Treatment of HeLa cells with 20 µM of Tripolin A for 5 h and 24 h, reduced the detected levels of pAurora A by 85% and 47% respectively (Figure 2A, 2B). Total Aurora A bound on the spindle was reduced by a similar percentage to pAurora A (81% and 24% after 5 h and 24 h respectively). Treatment with the previously reported Aurora A selective inhibitor MNL8237 [23] abolished the levels of pAurora A after 24 h of treatment, while levels of total Aurora A bound on the spindle were reduced by 70% (Figure S1A, S1B).

Tripolin B treatment, however, did not affect the levels of pAurora A in mitotic cells after 5 h of treatment, while longer treatment (24 h) unexpectedly, increased them significantly (by 40%). Total Aurora A bound on the spindle at similar levels to control cells (Figure 2A, 2B).

Aurora A protein levels, detected by Western blot 24 h post-treatment, were not significantly affected upon Tripolin A or B treatment (Figure 2C), or by MLN8237 (Figure S1C) indicating that Aurora A is not down-regulated or degraded in the presence of any of the compounds. Although the overall protein levels of Aurora A remained unaltered, the spindle- bound fraction of the protein was significantly reduced, upon Tripolin A and MLN8237 treatment, most likely due to an alteration of Aurora A recruitment on the spindle MTs. Therefore, the decreased pAurora A levels induced by Tripolin A indicate a reduction of Aurora A activity *in vivo* and not degradation of the protein.

In order to evaluate the selectivity of Tripolins for Aurora A over the structurally related Aurora B kinase, we performed Western Blot and immunofluorescence for the detection of phosphorylated Histone H3 on Ser-10, an Aurora B-specific substrate in cells. None of the Tripolins inhibited Histone H3 S10 phosphorylation, or altered Aurora B localization (Figure 2C, 2D).

Regarding Tripolin B, the experiments in HeLa cells cannot clarify whether binding of this compound leads to a genuine hyperphosphorylation of Aurora A, while they come in contrast to the *in vitro* results showing that Tripolin B binds and inhibits Aurora A kinase activity (Figure 1). Therefore, it was not pursued further in this study.

In conclusion, Tripolin A reduces the active fraction of Aurora A on the spindle, without affecting Aurora B, indicating that Tripolin A could act as an Aurora A inhibitor *in vivo*.

### Tripolin A induces mitotic spindle defects and spindle pole abnormalities

**Figure 3:**
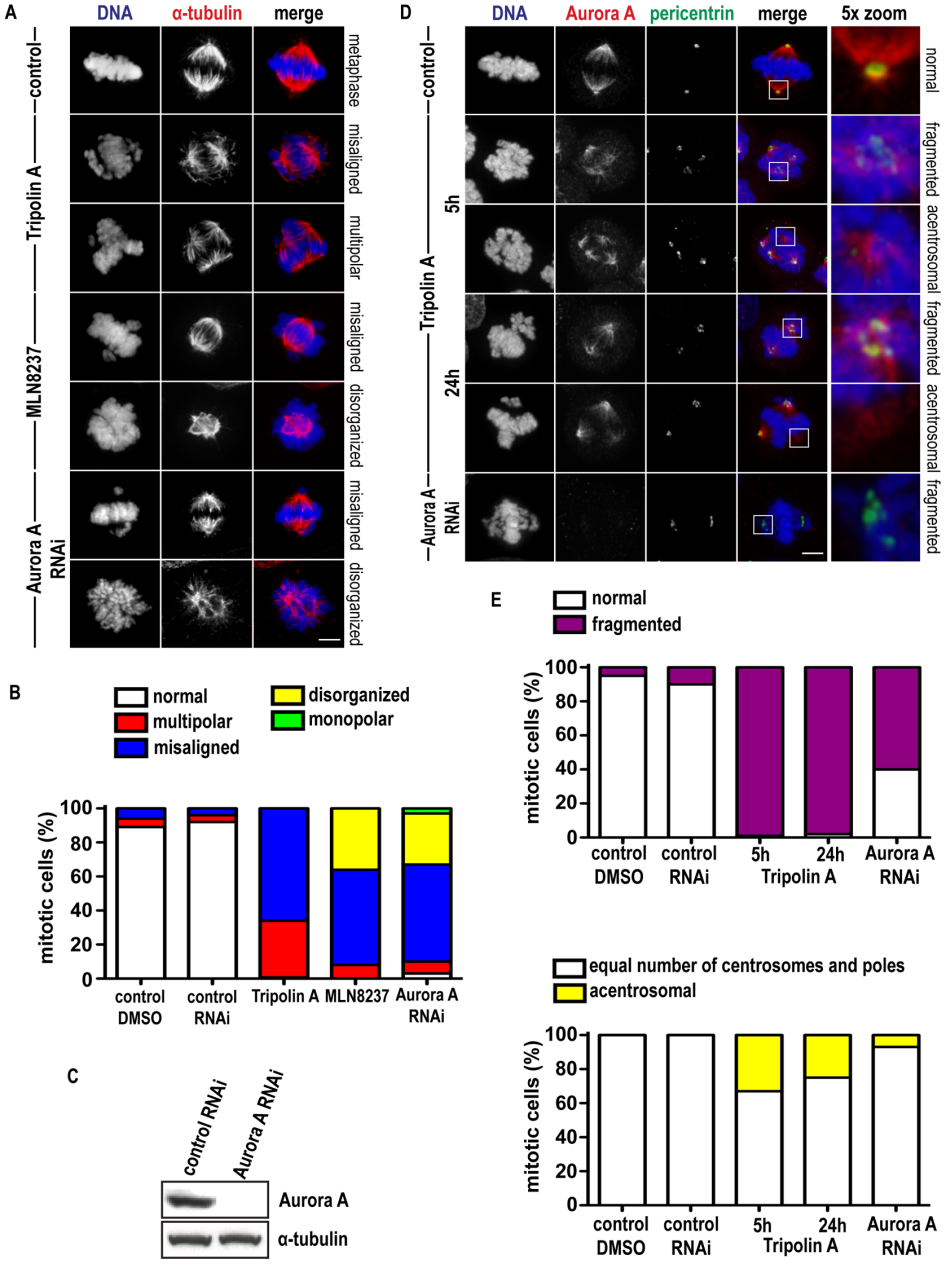
Tripolin A treatment results in spindle and centrosomal defects. (A) Representative immunofluorescence images of mitotic HeLa cells treated with DMSO, 20 µM Tripolin A for 24 h, 100 nM MLN8237 for 24 h or Aurora A siRNAs. In the merged images α-tubulin is pseudocolored red, DNA blue. (Scale bars, 5 µm). (B) Graph showing the percentage of normal, multipolar, misaligned, disorganized and monopolar figures in control mitotic cells (DMSO or control siRNAs) and mitotic cells treated with Tripolin A, MLN8237 or Aurora A siRNA (n = 300 cells for each group, from three independent experiments). (C) Western Blot analysis for Aurora A levels in Aurora A siRNA treated cells. α-tubulin was used as a loading control. (D) Images of mitotic HeLa cells treated with DMSO, 20 µM Tripolin A for 5 h and 24 h or Aurora A siRNA. In the merged images Aurora A is pseudocolored red, pericentrin green, DNA blue. (Scale bar 5 µm). (E) Graph showing the percentage of mitotic cells with fragmented centrosomes (up), or acentrosomal poles (down) in control mitotic cells (DMSO or control siRNA) and mitotic cells treated with Tripolin A, or Aurora A siRNA (n = 150 cells for each group, from three independent experiments).

Formation of abnormal mitotic spindles is consistent with Aurora A depletion by RNAi [21], [22], [31], or with treatment with specific Aurora A inhibitors (such as MLN8054 [21], [22]). The effect of Tripolin A on spindle organization and chromosome alignment was examined in HeLa cells by immunofluorescence. After 5 h of treatment the effect on spindle formation and chromosome alignment was so severe that no clear phenotype could be distinguished (Figure 2A). After 24 h of treatment, where partial recovery of the inhibition was observed, almost all cells (99.3%) showed mitotic defects, that could be distinguished into two categories: chromosome misalignment (66%), and aberrant spindle formation, mainly tripolar (33.3%) (Figure 3A, B). The DMSO-treated control cells displayed normal bipolar mitotic spindles with chromosomes properly aligned along the metaphase plate (Figure 3A, 3B). Treatment with the MNL8237 or with siRNAs against Aurora A also caused mainly chromosome alignment defects (56% for MLN8237 and 57% for Aurora A RNAi) as well as aberrant spindle formation (36% for MLN8237 and 30% for Aurora A RNAi) that was not possible to count number of poles (therefore termed disorganized) (Figure 3A, 3B, 3C).

Aurora A depletion by RNAi causes centrosome fragmentation [32]. To examine the effect of Tripolin A on centrosomes and spindle poles, mitotic HeLa cells were fixed 5 h or 24 h post-treatment and stained using pericentrin and γ-tubulin for centrosomes, and Aurora A and TPX2 for spindle poles. Control metaphase cells primarily (95%) possessed two centrosomes and two spindle poles per cell. Almost all mitotic cells treated with Tripolin A presented centrosome fragmentation (99% at 5 h and 98% at 24 h, Figure 3D, 3E), while Aurora A depletion by RNAi also caused severe centrosome fragmentation (60%, Figure 3D, 3E).

In addition, Tripolin A treated cells frequently (33% after 5 h and 25% after 24 h) formed acentrosomal spindle poles (Aurora A and TPX2 positive, pericentrin and γ-tubulin negative) forming three or more poles per cell, with centrosomal markers being absent/not detected in at least one of the poles (Figure 3D, 3E and Figure S2A). Radial arrays of MTs were emanating from all spindle poles, even the ones without centrosomal markers, indicating nucleation not originating from centrioles. Acentrosomal spindle formation was also observed to a lesser extend (7%) upon Aurora A depletion by RNAi (Figure 3C, 3D), and upon 5 h or 24 h treatment (12% and 10% respectively) with the Aurora A inhibitor MLN8237 (Figure S2B, S2C), while it has been reported to occur also upon treatment with another Aurora A selective inhibitor, the MLN8054 [22]. Since centrosome fragmentation as well as acentrosomal pole formation was apparent 5 h and 24 h post-treatment to a similar extend, the centrosomal abnormalities primarily occurred due to dysfunction of Aurora A and not as a consequence of an abnormal mitotic event in the presence of the compounds. Therefore, we conclude that Tripolin A induces mitotic defects specific to Aurora A inhibition.

### Tripolin A influences spindle size and MT organization

**Figure 4:**
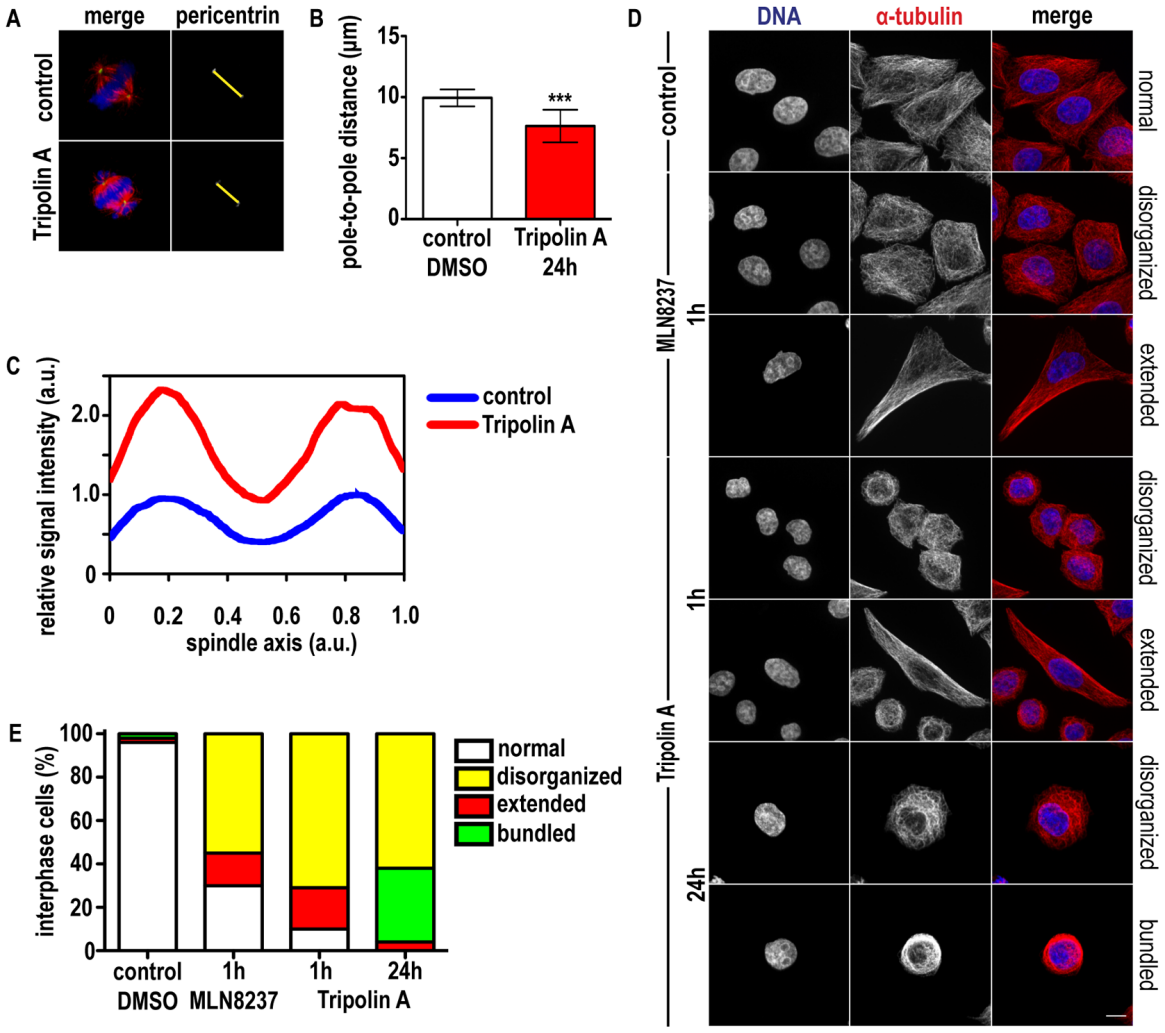
Tripolin A alters pole-to-pole distance and MT stability in mitotic cells and influences interphase MT array. (A) Maximum projections from z-stacks of a representative control cell and representative cells treated with Tripolin A. In the merged images α-tubulin is pseudocolored red; pericentrin is green, DNA is blue. Yellow arrows indicate interpolar distance. (B) Interpolar distances were measured based on pericentrin staining in HeLa cells (n≥100 cells for each group, from at least three independent experiments). ***: p<0.0001; (Student's t-test, two-tailed). Error bars indicate SD. (C) Longitudinal line scans of tubulin intensity from metaphase spindles of control and Tripolin A treated HeLa cells (n = 5 for each group). Intensities were normalized to the maximum value of the control curve, and spindle size was interpolated. Curves indicate mean values. (D) Representative immunofluorescence images of HeLa cells in interphase treated with DMSO, 100 nM MLN8237 for 1 h or 20 µM Tripolin A for 1 h and 24 h. In the merged images α-tubulin is pseudocolored red, DNA blue. (Scale bar 10 µm). (E) Graph showing the percentages of interphase cells with altered MT array, classified in the indicated arbitrary categories in control cells (DMSO) and cells treated with MLN8237 or Tripolin A (n = 150 cells for each group, from three independent experiments).

Since Aurora A activation by TPX2 is required for proper spindle length [33], we investigated the effect of Tripolin A on the interpolar distance measured in fixed samples stained with antibodies against α-tubulin and pericentrin. Cells treated with Tripolin A for 24 h had shorter mean pole-to-pole distance (7.6 µm±1.3, Figure 4A, 4B) compared to control cells (9.9 µm±0.7). Lack of Aurora A interaction with TPX2, which affects spindle-associated Aurora A but not centrosome-localized Aurora A [2], [33], has been reported to induce shorter spindles [33]. Tripolin A affects both spindle-associated and centrosomal-associated Aurora A (Figure 2A), therefore the shorter spindles observed upon Tripolin A treatment are consistent with the inhibition of the Aurora A kinase.

In order to test whether shorter spindles contained less MTs, we quantified MT intensities on the metaphase spindles. Cells treated with Tripolin A showed significantly increased fluorescent MT intensity along MTs. Longitudinal line scans of MT fluorescent intensity from metaphase spindles showed almost double MT intensity along the length of the MTs, compared to control cells, indicating more stable/bundled spindle MTs (Figure 4C). This finding is consistent with a recent observation that treatment of cells with the selective Aurora A inhibitor MLN8237 results in hyperstable spindles [34].

Since it has been shown that Aurora A kinase modulates dynamic instability of interphase MT while Aurora B does not [35], [36] we also explored the effect of Tripolin A in interphase. Following treatment with Tripolin A, the organization of the interphase network MT was extensively modified and presented abnormalities that were arbitrarily classified into three categories: disorganized, elongated/extended and bundled network (Figure 4D). Cells treated with Tripolin A for 1 h exhibited mainly shorter and disorganized or locally extended MT network similarly to the MLN8237 treatment (Figure 4D, 4E) and consistent with the effect of other Aurora A specific compounds [35]. Longer exposure to Tripolin A (24 h) further modified the MT network inducing more severe MT disorganization where cells appeared to have an entirely collapsed MT array and were classified as bundled (Figure 4D, 4E). Thus Tripolin A affects MT dynamics both in mitosis and interphase, in a manner similar to specific Aurora A inhibitors.

### Tripolin A affects the precise localization of HURP

HURP is an Aurora A substrate [7], required for chromatin-dependent MT nucleation, localizing preferentially to regions of kt-MTs and affecting their stability [9], [10], [37].

**Figure 5:**
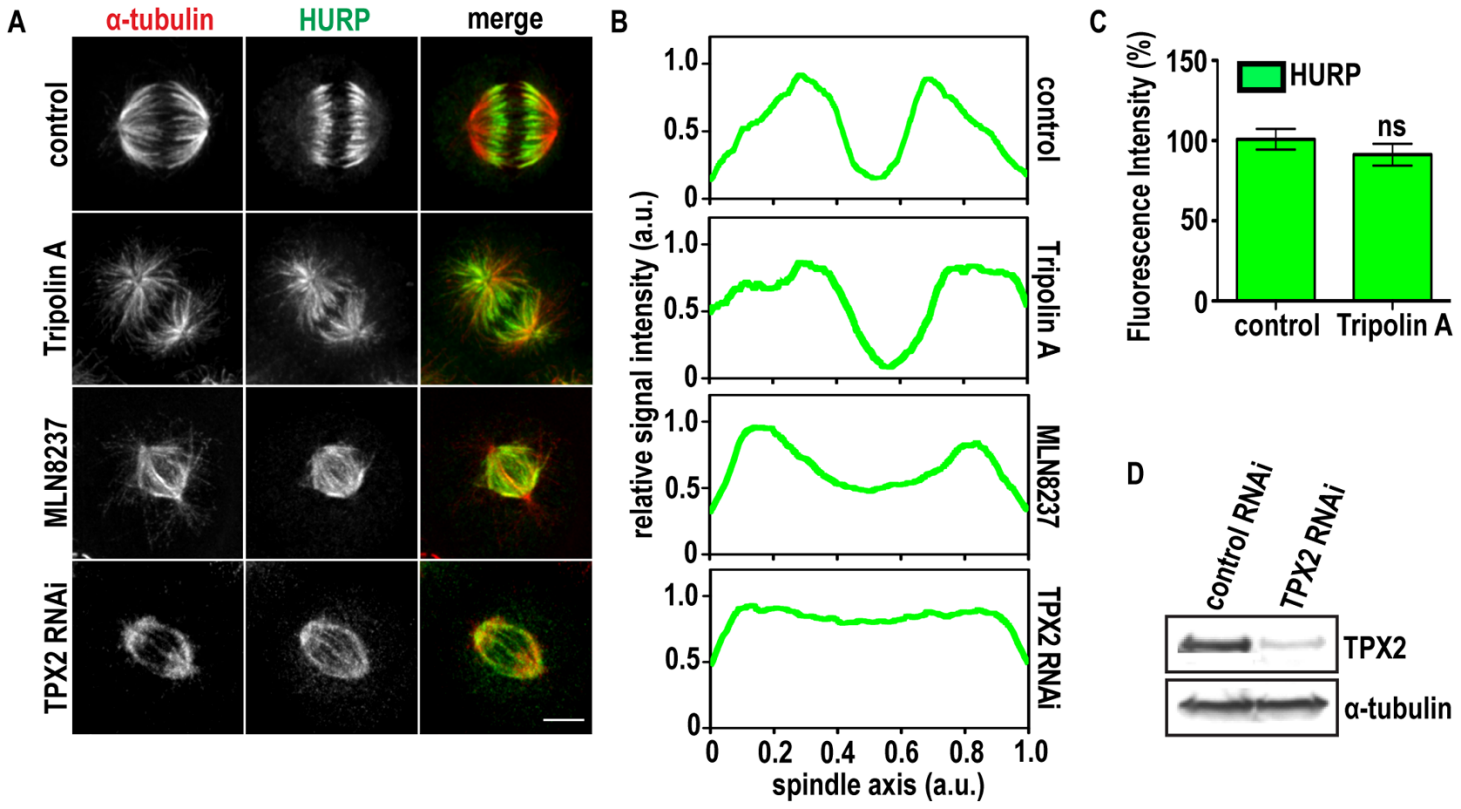
Inhibition of Aurora A alters the localization of HURP. (A) Representative immunofluorescence images of metaphase cells treated with DMSO, 20 µM Tripolin A or 100 nM MLN8237 for 24 h, or TPX2 siRNAs. In the merged images α-tubulin is pseudocolored red, HURP green. (Scale bars, 5 µm). (B) Longitudinal line scans of HURP intensity from metaphase spindles of control and Tripolin A treated HeLa cells (n = 5 for each group). Intensities were normalized to maximum value within the same spindle, and spindle size was interpolated. Curves indicate mean values. (C) Fluorescence intensity (% percentage) of HURP quantified in control metaphase cells and cells treated with Tripolin A (n≥20 cells for each group, from at least two independent experiments). ns p>0.05; (Mann-Whitney test, two-tailed). Error bars represent SEM. (D) Western Blot analysis for TPX2, in TPX2 siRNAs treated cells. α-tubulin was used as a loading control.

It has been suggested that HURP's binding on MTs is regulated by Aurora A phosphorylation [8], [12], therefore we tested the effect of Tripolin A on HURP localization at metaphase spindles. HURP's binding on MTs was not significantly affected upon Tripolin A treatment (Figure 5A, 5C). Instead, treated cells exhibited a change in the distribution pattern of HURP on the spindle MTs. Longitudinal line scans of HURP's fluorescent intensity from metaphase spindles in control-treated cells showed maximal levels of the protein in the vicinity of chromosomes (Figure 5A, 5B), consistent with the fact that HURP is a Ran-GTP regulated protein [9], [10], [37]. In contrast, Tripolin A treated cells exhibited more HURP signal towards the poles. To examine the specificity of Aurora A effect on HURP's distribution on MTs, we analyzed its distribution in cells treated with the selective Aurora A inhibitor MLN8237, and in cells were spindle-associated Aurora A was abrogated by TPX2 depletion (Figure 5A, 5B, 5D). Both treatments caused miss-localization of HURP and loss of its gradient MT binding towards the chromosomes. However, the phenotypes observed upon these treatments, although similar, were not identical to Tripolin A treatment, and this could be attributed to the differential way they affect Aurora A activity or localization.

Therefore, the selective binding of HURP on spindle MTs in the vicinity of chromosomes was altered when Aurora A activity or localization were affected. These results indicate that HURP's phosphorylation by Aurora A is not required for its direct binding on the MTs, but rather for its precise localization and distribution along spindle MTs.

## Discussion

Here we describe the development and evaluation of a novel Aurora A kinase inhibitor, named Tripolin A, as well as its effect on certain mitotic MAPs. Although two chemically similar small-molecules could inhibit Aurora A kinase activity *in vitro*, only Tripolin A showed specific inhibition of Aurora A with no significant effect on Aurora B, in mammalian cells.

Tripolin A treatment recapitulated phenotypes associated with RNAi and chemical inhibition of Aurora A, including centrosome integrity, spindle formation and length, as well as MT organization in interphase. Additionally, Tripolin A interfered with the precise distribution of HURP, a substrate of the Aurora A kinase, on spindle MTs.

HURP shows a gradient localization towards the chromosomes, which is exquisitely sensitive to RanGTP levels while it is not affected by altered MT dynamics [10]. By using single-cell microscopy quantification analysis we were able to evaluate delicate alterations in protein localization that would not be apparent using conventional population studies. It has been suggested that HURP's binding on MTs is regulated through phosphorylation by Aurora A [8]. However, Aurora A depletion by siRNA (Figure S3) or mutation of the potential Aurora A phosphorylation residues on HURP [12], did not prevent HURP's binding on the MTs. Here we show that altering the levels or the localization of Aurora A resulted in loss of the gradient localization pattern of HURP in the proximity of the chromosomes, indicating a spatial regulation of HURP's distribution on the metaphase MTs. Consistently, a GFP-fused N-terminal fragment of HURP that lacks the C-terminus where Aurora A phosphorylation occurs, shows a distribution closer to the spindle poles and away from the chromosomes, resembling the effect of Tripolin A treatment (our unpublished observations, [8], [38]). Therefore it is likely that Aurora A kinase regulates the spatial distribution of HURP on MTs, with a positive gradient towards the chromosomes, rather than its MT binding *per se*.

An understanding of the role Aurora A plays in regulating the MT network that forms the spindle is emerging. In one model Aurora A is critical for the regulation of the EXTAH multiprotein complex, comprised of Eg5, XMAP215, TPX2, Aurora A, and HURP, which have MT binding, cross-linking, and kinesin motor activities. Together they act to bundle, cross-link, and stabilize the growing MT network. Disruption of any component of the complex perturbs spindle formation [9]. In this context, HURP is affecting primarily the stability of kt-MTs [9], [11], due to its proximity to the chromosomes. Disruption of the gradient distribution and improper localization of HURP on the spindle poles upon Aurora A perturbation, most likely will alter MTs stability and disturb the balance which leads to proper spindle formation.

It is interesting to note that the below treatments: a. loss of spindle-associated Aurora A through TPX2 depletion, b. inhibition of active Aurora A by the selective inhibitor MLN8054 (our unpublished observations) or the second-generation MLN8237, or c. inhibition by Tripolin A, all cause loss of HURP's gradient distribution.

MLN8054 is a first generation ATP-competitive Aurora A selective inhibitor, and molecular dynamics studies showed that this selectivity is due to the induced changes in the conformation of the activation loop of the kinase, forcing it to adopt an unusual DFG-up conformation [39]. Tripolin A showed non-ATP competitive mode of action *in vitro*. Docking analysis indicated that it could bind and/or stabilize the inactive forms of Aurora A either via the deep back pocket present in the DFG-out conformation of inactive Aurora A, or with a lower probability, via the small hydrophobic side-pocket of the DFG-up conformation (Figure S4 and Supporting Information S1). Therefore Tripolin A could bind or stabilize a different conformation of inactive Aurora A kinase compared to MLN8054. Taken together, even though Tripolin A does not have a very high affinity for the Aurora A kinase, nonetheless it has a value as a compound that does not have preference for binding at the ATP binding pocket and could serve as a scaffold for the development of specific and higher affinity Aurora A inhibitors.

Small-molecule manipulation of protein kinases is a powerful tool for studying the biological context in which they function. When kinases are assayed *in vitro* in isolation from their physiological partners, screens cannot accurately mimic the complex environment under which these compounds function *in vivo*. Considering the diversity of the pathways in which Aurora A participates, targeting particular active or inactive DFG conformations, or certain Aurora A-containing sub-complexes may in the future become a preferable approach. Regardless of whether such Aurora A inhibitors will succeed in cancer therapy, they represent a potent tool to tease apart the effects of Aurora A inhibition.

## Experimental Procedures

### Chemical Synthesis

#### General procedures

^1^H and ^13^C NMR spectra were recorded either on a Varian GEMINI 300 or Varian GEMINI 200 spectrometer at room temperature. Mass spectra were measured on a Finnigan MAT MS 70 (EI) spectrometer or on a Bruker Daltonics Apex II (ESI). Melting points are uncorrected.

**Figure 6:**
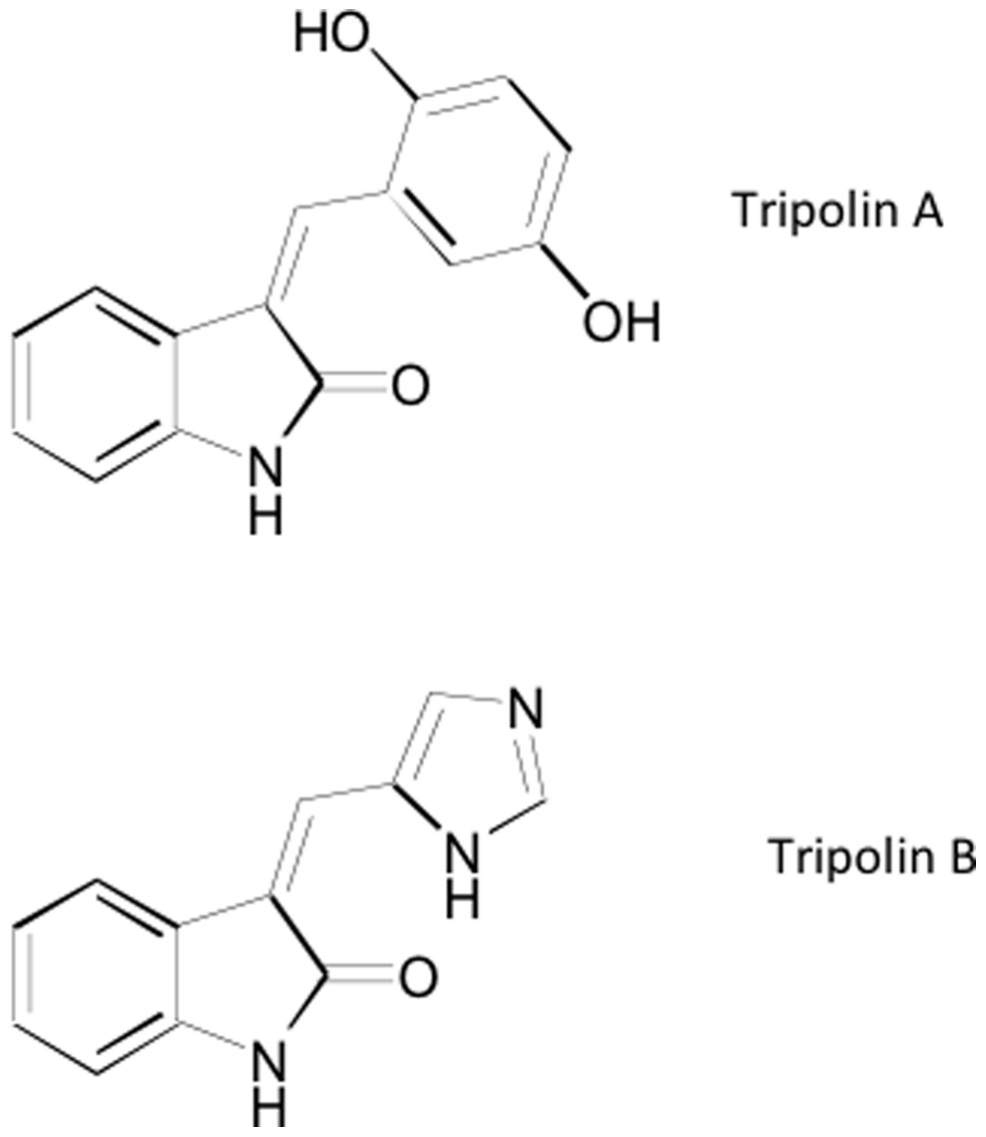
Chemical structure of Tripolin A and Tripolin B.

Syntheses were performed as described previously [40]. Tripolin A and Tripolin B are shown in Figure 6.

### 3-(2,5-Dihydroxy-benzylidene)-1,3-dihydro-indol-1-one (Tripolin A)

Yield = 52%; mp 256°C; ^1^H-NMR (300 MHz, (CD_3_)_2_SO) δ = 6.77–6.94 (m, 4H, 4x C*H*_arom._); 7.10 (d, *^4^J* = 2.7 Hz, 1H, C*H* = ); 7.24 (t, *^4^J* = 7.5 Hz, 1H, C*H*_arom._); 7.64–7.70 (m, 2H, 2x C*H*_arom._); 9.11 (br, 1H, O*H*); 9.43 (br, 1H, O*H*); 10.58 (br, 1H, N*H*); ^13^C-NMR ((CD_3_)_2_SO, 75.5 MHz) δ = 110.64, 115.52, 117.56, 119.68, 121.73 (5x *C*H_arom._), 121.96 (*C*_q._), 122.20 (*C*_q._), 123.35 (*C*H_arom._), 126.77 (*C*_q._), 130.33 (*C*H_arom._), 133.40 (*C*H = ), 143.31 (*C*_q._), 150.12 (*C*_q._-OH), 150.15 (*C*_q._-OH), 169.66 (*C*O); HR-MS (ESI, MeOH) m/z for C_15_H_12_NO_3_ [M+H]^+^: calculated: 254.08117, found: 254.08136.

### 3-(3*H*-Imidazol-4-ylmethylene)-1,3-dihydro-indol-2-one (Tripolin B)

Yield = 88%; mp 253°C; ^1^H-NMR (200 MHz, (CD_3_)_2_SO) δ = 6.87–7.03 (m, 2H, 2x C*H*_arom._); 7.15–7.19 (m, 1H, C*H*_arom._), 7.63–7.81 (m, 2H, C*H*_arom._, C*H* = ); 7.82 (s, 1H, C*H* = ); 8.01 (s, 1H, C*H* = ); 11.06 (br, 1H, N*H*); 13.73 (br, 1H, N*H*);

^13^C-NMR (50.3 MHz, (CD_3_)_2_SO) δ = 109.67, 119.19 (2x *C*H_arom._), 120,26 (*C*_q._), 121.37 (*C*H_arom._), 123.42 (*C*H = ), 124.45 (*C*_q._), 127.94 (*C*H_arom._), 129.17 (*C*_q._), 139.02 (*C*H_arom._), 139.07 (*C*H_arom._), 139.76 (*C*_q._), 168.75 (*C*O);

HR-MS (ESI, MeOH) m/z: for C_12_H_10_N_3_O [M+H]^+^: calculated: 212.08184, found: 212.08204.

### Protein Expression and Purification

Aurora A full length was subcloned into the pET21d (Novagen) vector. The recombinant Aurora A-6xHis protein was expressed in *E.coli* strains and purified under non-denaturing conditions via Ni-NTA metal affinity HiTrap Chelating HP column (Amersham Biosciences).

### *In vitro* kinase assays

*In vitro* kinase assays were performed at the Chemical facility of the European Molecular Biology Laboratory (EMBL), as previously described [24]. The IC_50_ values for the different compounds were determined by using the Luminescence ATP Detection Assay System for Kinase Applications, Easylite-kinase (Perkin Elmer), as well as the Z'LYTE Kinase Assay Kit-Ser/Thr 1 Peptide PV3174 (Invitrogen) by following the manufacturer's instructions. The ATP competition assays and the kinase selectivity profile were assessed using the Z'LYTE Kinase Assay.

### Differential Scanning Fluorimetry (DSF)/Protein Stability Shift assay

Thermal stability experiments were carried out using the 7500 Real Time PCR System (Applied Biosystems). Aurora A-6xHis protein (5 µM) was assayed in 20 µl of PBS1x, 150 mM NaCl, 2 mM MgCl_2_, 8.7% glycerol, pH 6.5 in a 96-well plate. Tripolins A and B were added at a final concentration of 100 µM. SYPRO Orange (1∶1000; Invitrogen) was added as a fluoresence probe. Appropriate excitation and emission filters for the SYPRO-Orange dye were set. The temperature was raised at 1°C/min from 26°C to 80°C and fluorescence readings were taken at each interval. Data acquisition was performed using the SDS Software version 1.4. Data analysis and plotting was performed in GraphPad Prism® Version 5.0a software.

### Cell culture, Immunofluorescence and Western Blot analysis

HeLa cells were grown in DMEM supplemented with 10% FBS, 2 mM L-glutamine, 100 U/ml penicillin, and 100 µg/ml streptomycin (Invitrogen) at 37°C with 5% CO_2_ in a humidified incubator. For immunofluorescence staining, cells grown on No.1 glass coverslips were fixed in 3.7% formaldehyde/PHEM (60 mM PIPES, 25 mM Hepes, 10 mM EGTA, 2 mM MgCl_2_) pH 6.9 for 20 min at 37°C and then permeabilized in PBS/0.1% v/v Triton X-100 pH 7.4 for 5 min at room temperature or were fixed/permeabilized in −20°C methanol for 3 min. Cells were blocked in PBS/5% w/v BSA pH 7.4 and stained with various combinations of: anti-Aurora A pT288 rabbit polyclonal antibody (1∶100; Cell Signaling Technology), anti-Aurora A mouse monoclonal antibody (1∶2000; Abcam), anti-AIM-1/Aurora B mouse monoclonal antibody (1∶500; BD Biosciences), anti-pHistoneH3 (Ser10) rabbit polyclonal antibody (1∶1000; Millipore), anti-pericentrin rabbit polyclonal antibody (1∶2000; abcam), anti-α-tubulin mouse monoclonal antibody clone GTU-88 (1∶500; Sigma-Aldrich), anti-α-tubulin mouse monoclonal antibody (1∶1000; Santa Cruz Biotechnology), rabbit polyclonal antisera against HURP and TPX2 [9], [41], for 1 h at room temperature. Cells were washed in PBS pH 7.4, incubated with appropriate Alexa Fluor 488 and 568 secondary antibodies (1∶500; Molecular Probes) for 30 min a room temperature and DNA was counterstained with DAPI (1 µg/ml; AppliChem). After final washes coverslips were mounted in homemade mowiol mounting medium.

For Western blot analysis of compound treated cells, HeLa cells were arrested with thymidine (2 mM) for 18 h, released into fresh medium for 6 h, and blocked with nocodazole (60 ng/ml) for 20 h. DMSO or the different compounds were added 2 h after thymidine release. Mitotic cells were shaken off and lysed in RIPA buffer 50 mM Tris pH 8, 150 mM NaCl, 50 mM sodium orthovanadate, 1% v/v NP40, 0,1 mM PMSF supplemented with complete protease inhibitors cocktail (Roche). For western blot analysis of asynchronous treated cells (siRNA treatment), cells were washed twice with ice cold PBS and lysed in RIPA buffer.

The protein extract for both cases (30 µg; as determined by the Bradford assay, Bio-Rad) was loaded on SDS-PAGE, transferred to a nitrocellulose membrane and probed with anti-Aurora A mouse monoclonal antibody (1∶1000; Abcam), anti-AIM-1/Aurora B mouse monoclonal antibody (1∶1000; BD Biosciences), anti-pHistoneH3 (Ser10) rabbit polyclonal antibody (1∶1000; Millipore), anti-α-tubulin mouse monoclonal antibody (1∶1000; Santa Cruz Biotechnology), TPX2 [41] (1∶1000).

### Compound treatment and RNAi

HeLa cells were treated for 1 h, 5 h or 24 h with 20 µM Tripolin A, 20 µM Tripolin B or 100 nM MLN8237 (SelleckBio) diluted in DMSO while cells treated with DMSO (0.1% v/v) served as control, unless other wise stated.

siRNAs against TPX2 5′-GGGCAAAACTCCTTTGAGA-3′ [33] and Aurora A

5′-ATGCCCTGTCTTACTGTCA-3′ [2] were purchased from Ambion. siRNA control was also purchased from Ambion (Silencer Negative Control #1 siRNA). 24 h hours after plating cells were transfected with 100 nM siRNA duplexes prepared in OptiMEM Reduced Serum Medium (Gibco) using Lipofectamine 2000 (Invitrogen). Growth medium containing transfection complexes was replaced with fresh complete medium 6 hours after the transfection. Cells were assayed 24 h post-transfection for TPX2 RNAi and 48 h post transfection for Aurora A RNAi.

### Microscopy and Image analysis

Imaging of fixed samples was performed on a customized Andor Revolution Spinning Disk Confocal System built around a stand (IX81; Olympus) with a 100x-1.4 NA lens and a digital camera *(*Andor Ixon+885) (CIBIT Facility, MBG-DUTH) or on a Zeiss LSM780 laser scanning confocal microscope (ALMF-EMBL). Image acquisition was performed in Andor IQ 1.10.3 software or in Zen 2010 respectively. Optical sections were recorded every 0.3 µm.

Image intensity analysis for data sets was performed in ImageJ 1.44n (National Institute of Health, USA) software where image-processing macros were developed. The two-dimensional (2D) average projection of z-stack images were quantified after background subtraction for Aurora A, TPX2, HURP α-tubulin using a fixed size cycle area where integrated intensity values were measured. For Aurora A, TPX2 and HURP quantification appropriate threshold was set in order to quantify only the on spindle signal. For pAurora A T288 intensity quantification, a thresholding-segmentation method [42] was performed to define centrosomal area according to pAurora A T288 signal on 2D average projections of z-stack images and integrated intensities were measured. Interpolar distances were measured on 2D maximum projections of z-stack images using the analysis tools of the image acquisition software Andor IQ 1.10.3. Statistical Analysis and plotting was performed using the GraphPad Prism Version 5.0a software. All microscopy images presented here are 2D maximum intensity projections of z-stack images (ImageJ 1.44n National Institute of Health, USA).

Linescans were generated after background subtraction in average 2D projection images, by manually drawn lines (1.5 microns in thickness) from pole to pole in bipolar metaphase cells which was marked by Aurora A or α-tubulin signal (ImageJ 1.44n National Institute of Health, USA). X and Y values were normalized against maximum values in the same cell, therefore, are expressed in arbitrary units. To compare intensities of spindles varying in size, we interpolated the data to identical length intervals (GraphPad Prism Version 5.0a software).

## Supporting Information

Figure S1 **Effect of MLN8237 on Aurora A.** (A) Representative immunofluorescence images of HeLa cells in metaphase, treated with solvent control (DMSO) or 100 nM MLN8237 for 24 h. In the merged images Aurora A is pseudocolored red, pAurora A T-288 green, DNA blue. (Scale bars 5 µm). (B) Fluorescence intensity (% percentage) of total Aurora A on spindle was quantified in control metaphase cells and cells treated with MLN8237 (n≥20 cells for each group, from at least two independent experiments). ***: p<0.001; (Mann-Whitney test, two-tailed). Error bars represent SEM. (C) Western Blot analysis for Aurora A in control and MLN8237 treated cells. α-tubulin was used as a loading control.
(TIF)

Figure S2 **Effects of Tripolin A and MLN8237 on centrosome organization.** (A) Representative immunofluorescence images of HeLa cells in metaphase, treated with solvent control (DMSO) or 20 µM Tripolin A for 24 h. In the merged images TPX2 is pseudocolored red, γ-tubulin green, DNA blue. (Scale bars 5 µm). (B) Images of mitotic HeLa cells treated with solvent control (DMSO) or 100 nM MLN8237 for 5 h and 24 h. In the merged images Aurora A is pseudocolored red, pericentrin green, DNA blue. (Scale bar 5 µm). (C) Graphs showing the percentage of mitotic cells with fragmented centrosomes (up), or acentrosomal poles (down) in control mitotic cells (DMSO) and mitotic cells treated with MLN8237 for 5 h and 24 h. (n = 150 cells for each group, from three independent experiments).
(TIF)

Figure S3 **Aurora A depletion by siRNA does not affect MT binding of HURP.** Fluorescence intensity (arbitrary units) of HURP bound on spindle MTs was quantified in control and Aurora A depleted metaphase cells (n≥20 cells for each group, from at least two independent experiments). ***: p<0.001; ns: p>0.05; (Mann-Whitney test, two-tailed). Error bars represent SEM.
(TIF)

Figure S4 ***In silico*** **recognition of Aurora A by Tripolin A.** Docking analysis of Tripolin A was conducted using Aurora A crystal structures from complexes with ADP-TPX2 (DFG-in, PDB code 1OL5), anilinopyrimidine (DFG-up, PDB code 3H10) and quinazoline-13 (DFG-out, PDB code 2C6E), which are shown in a wiremesh representation. Representative Tripolin A poses from clusters with highest P-value are shown in sticks (green, best scoring cluster; magenta, 2^nd^ best cluster). Arrowheads: green, ATP-binding pocket; sky-blue, deep pocket; white, putative secondary pocket. Parts of the glycine-rich loop (Gly-loop) and activation loop (A-loop) are also shown. Parts of protein surface are omitted for clarity.
(TIF)

Supporting Information S1 (DOC)
