# Supplementary figures and images for: Tripolin A, a Novel Small-Molecule Inhibitor of Aurora A Kinase, Reveals New Regulation of HURP's Distribution on Microtubules
Source: PLoS One. 2013 Mar 13;8(3):e58485. doi: 10.1371/journal.pone.0058485 (PMC3596387; doi:10.1371/journal.pone.0058485)

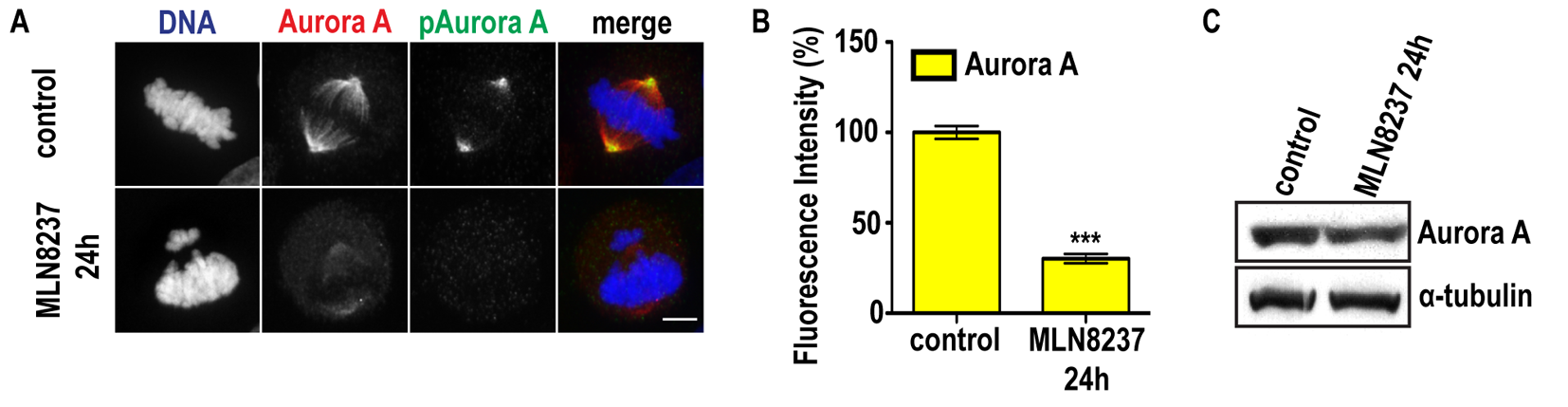

Supplement: Figure S1 — Effect of MLN8237 on Aurora A. (A) Representative immunofluorescence images of HeLa cells in metaphase, treated with solvent control (DMSO) or 100 nM MLN8237 for 24 h. In the merged images Aurora A is pseudocolored red, pAurora A T-288 green, DNA blue. (Scale bars 5 µm). (B) Fluorescence intensity (% percentage) of total Aurora A on spindle was quantified in control metaphase cells and cells treated with MLN8237 (n≥20 cells for each group, from at least two independent experiments). ***: p<0.001; (Mann-Whitney test, two-tailed). Error bars represent SEM. (C) Western Blot analysis for Aurora A in control and MLN8237 treated cells. α-tubulin was used as a loading control. (TIF) [file pone.0058485.s001.tif]

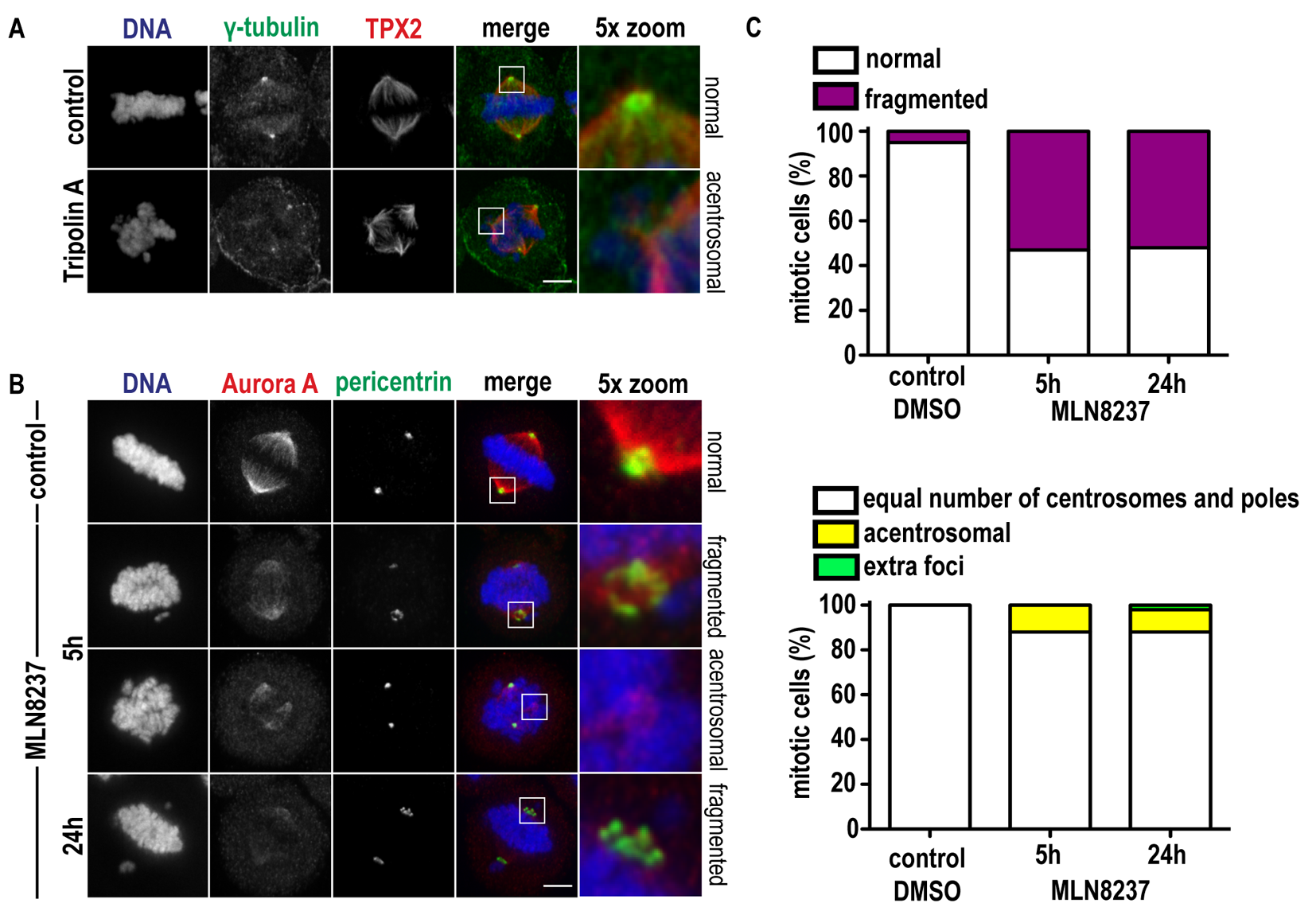

Supplement: Figure S2 — Effects of Tripolin A and MLN8237 on centrosome organization. (A) Representative immunofluorescence images of HeLa cells in metaphase, treated with solvent control (DMSO) or 20 µM Tripolin A for 24 h. In the merged images TPX2 is pseudocolored red, γ-tubulin green, DNA blue. (Scale bars 5 µm). (B) Images of mitotic HeLa cells treated with solvent control (DMSO) or 100 nM MLN8237 for 5 h and 24 h. In the merged images Aurora A is pseudocolored red, pericentrin green, DNA blue. (Scale bar 5 µm). (C) Graphs showing the percentage of mitotic cells with fragmented centrosomes (up), or acentrosomal poles (down) in control mitotic cells (DMSO) and mitotic cells treated with MLN8237 for 5 h and 24 h. (n = 150 cells for each group, from three independent experiments). (TIF) [file pone.0058485.s002.tif]

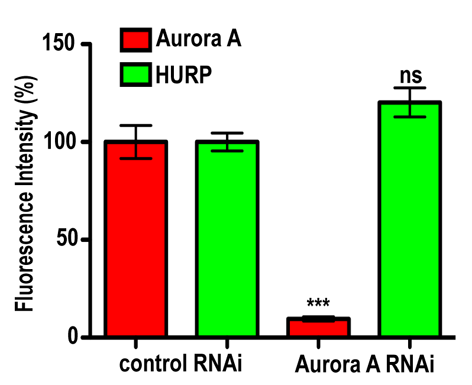

Supplement: Figure S3 — Aurora A depletion by siRNA does not affect MT binding of HURP. Fluorescence intensity (arbitrary units) of HURP bound on spindle MTs was quantified in control and Aurora A depleted metaphase cells (n≥20 cells for each group, from at least two independent experiments). ***: p<0.001; ns: p>0.05; (Mann-Whitney test, two-tailed). Error bars represent SEM. (TIF) [file pone.0058485.s003.tif]

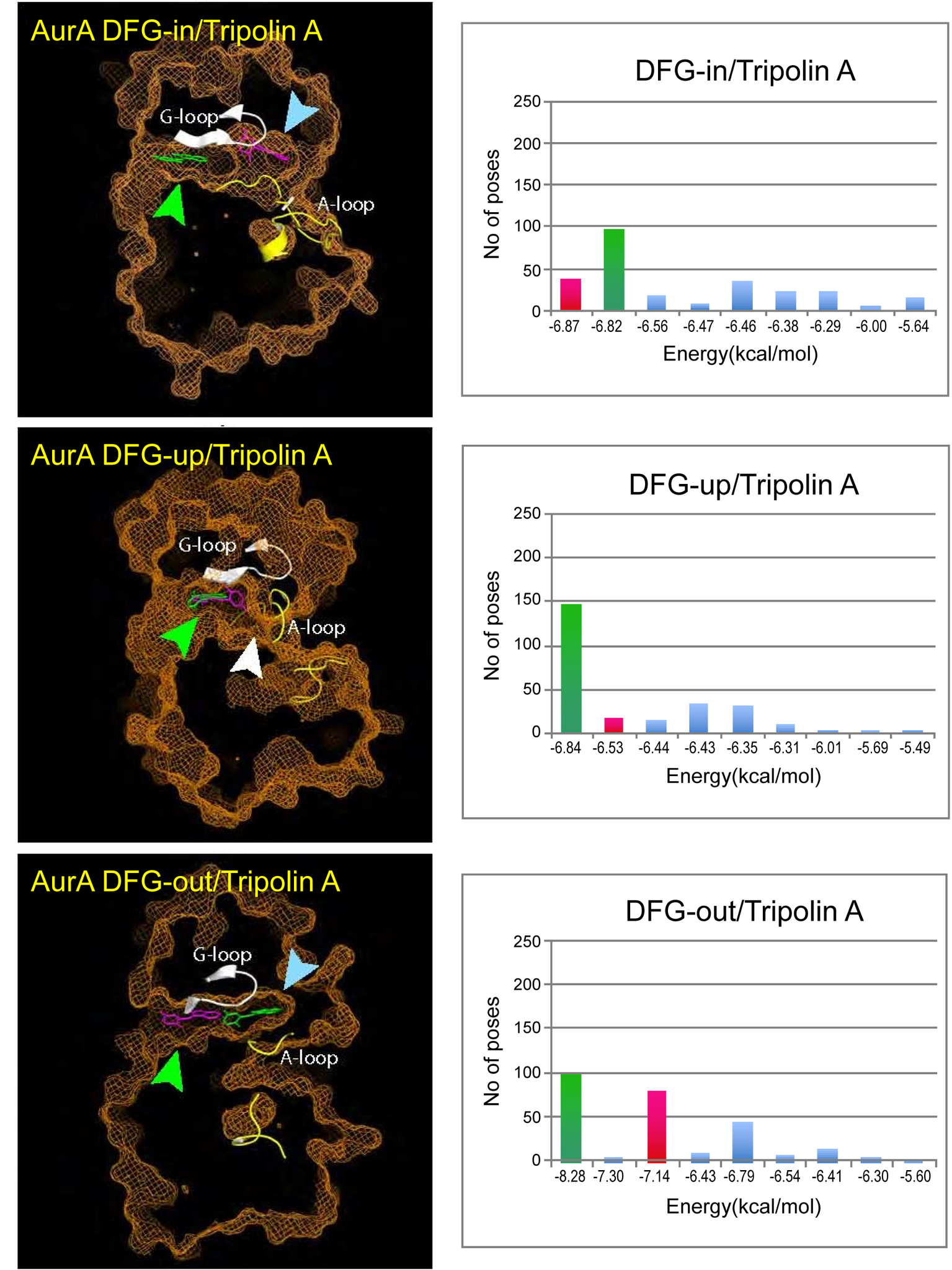

Supplement: Figure S4 — In silico recognition of Aurora A by Tripolin A. Docking analysis of Tripolin A was conducted using Aurora A crystal structures from complexes with ADP-TPX2 (DFG-in, PDB code 1OL5), anilinopyrimidine (DFG-up, PDB code 3H10) and quinazoline-13 (DFG-out, PDB code 2C6E), which are shown in a wiremesh representation. Representative Tripolin A poses from clusters with highest P-value are shown in sticks (green, best scoring cluster; magenta, 2nd best cluster). Arrowheads: green, ATP-binding pocket; sky-blue, deep pocket; white, putative secondary pocket. Parts of the glycine-rich loop (Gly-loop) and activation loop (A-loop) are also shown. Parts of protein surface are omitted for clarity. (TIF) [file pone.0058485.s004.tif]
