# Supplementary material for: Tripolin A, a Novel Small-Molecule Inhibitor of Aurora A Kinase, Reveals New Regulation of HURP's Distribution on Microtubules
Source: PLoS One. 2013 Mar 13;8(3):e58485. doi: 10.1371/journal.pone.0058485 (PMC3596387; doi:10.1371/journal.pone.0058485)
Supplement: Supporting Information S1 — (DOC) [file pone.0058485.s005.doc]

**Supporting Information**

***In silico* docking**

For Tripolin A docking simulation three distinct Aurora A crystal structures were used as target (stripped from ligands), each from Aurora A complexes characteristic of the three distinct activation loop conformations, DFG-in active (PDB: 1OL5), DFG-out inactive (PBD: 2C6E) and DFG-up inactive Aurora A conformation respectively (PDB: 3H10). The molecular docking program AutoDock (version 4.0) running a Lamarckian genetic algorithm (LGA) search method was used. Ligand structures were constructed using Accelrys DS. Target preparation, scoring, energy ranking and clustering were as implemented in AutoDock. The number of docking runs per simulation were 256, the maximum number of energy evaluations 2.5 mil., the mutation rate 0.02, the crossover rate 0.8 and the population size 150. Appropriate grid maps were constructed with a grid point spacing of 0.375 Å. To compare binding preference to various Aurora-A pockets, a probability function (P-value) for each pose was calculated according to . Higher P-values attribute higher confidence for the pose and are used here as a measure of binding specificity. Best binders are poses with the highest P-value that belong to distinct 3D clusters created using a 1.5 Å cutoff. Visual inspection of poses was performed in O and ligand-target interactions were analyzed using LIGPLOT. Figures were created with Pymol.

**Supporting Information references**

1. Sotriffer CA, Winger RH, Liedl KR, Rode BM, Varga JM (1996) Comparative docking studies on ligand binding to the multispecific antibodies IgE-La2 and IgE-Lb4. J Comput Aided Mol Des 10: 305-320.
